# The Alteration of HDL in Patients with AMI Inhibited Angiogenesis by Blocking ERK1/2 Activation

**DOI:** 10.1155/2022/1057772

**Published:** 2022-08-16

**Authors:** Wei Zhang, Zhe Li, Wen-Qi Han, Qun-Rang Wang, Hao-Yu Wu, Xin-Hong Liu, Kun Xing, Gong Cheng, Feng-Jun Chang

**Affiliations:** ^1^Department of Cardiology, Shaanxi Provincial People's Hospital, Xi'an, China; ^2^Department of Cardiology, Affiliated Hospital of Shaanxi University of Chinese Medicine, Xian'yang, China

## Abstract

**Objective:**

High-density lipoprotein (HDL) was found vasoprotective, but numbers of patients with acute myocardial infarction (AMI) have normal or even high levels of pathological HDL (pHDL). So, we investigate the mechanism of pHDL in AMI patients on angiogenesis.

**Methods:**

HDL with normal levels from healthy subjects (nHDL, control group, *n* = 20) and patients with AMI (pHDL, experimental groups, *n* = 30) were obtained by super high speed centrifugation. Then, effects of HDL on proliferation, migration, angiogenesis, and expression of ERK1/2 and its phosphorylation in human umbilical vein endothelial cells (HUVEC) with or without PD98059 (inhibitor of ERK1/2) preincubation were detected.

**Results:**

Compared with the control group (nHDL), HDL from the experimental group (pHDL) significantly inhibited the phosphorylation of ERK1/2, proliferation, migration, and angiogenesis of HUVEC (*P* < 0.05), while these effects of HDL could substantially be blocked by preincubation of PD98059 (*P* < 0.05).

**Conclusion:**

HDL in AMI patients affects angiogenesis by inhibiting ERK1/2 activation free from HDL levels.

## 1. Introduction

Normal high-density lipoprotein (nHDL) can be antiatherosclerosis and reduce the incidence of adverse cardiovascular events by its reversing cholesterol transport (RCT) from the blood to the liver [1, 2]. What is more, much more studies found that nHDL own the following functions: protecting endothelial function, anticoagulant, anti-inflammatory, antioxidant, and promoting angiogenesis beside RCT [3]. Angiogenesis and the effective establishment of collateral circulation are the main methods to restore coronary blood flow, which could reduce and prevent further myocardial damage after myocardial infarction [4]. Angiogenesis is a complex multistep process, including endothelial cell migration, proliferation, and differentiation into blood vessels. This process also involves the participation of various growth factors and signal pathways [5]. However, in the condition of disease, such as diabetes, chronic renal insufficiency, and coronary atherosclerotic heart disease, pathological HDL (pHDL) lost its protective effect and became proinflammatory, because the tyrosine in its functional protein apolipoprotein A-I is more easily modified by myeloperoxidase [6]. Our recent studies have found that the vasoprotective effects of pHDL on patients with valvular heart disease and those who undergo cardiac surgery are weaker than nHDL [7]. However, the mechanism of pHDL inhibiting angiogenesis in patients with coronary heart disease was unclear. In the present study, HDL (normal level) from acute myocardial infarction (AMI) patients was obtained, and its effects on angiogenesis were tested.

## 2. Materials and Methods

### 2.1. Study Population

AMI patients (*n* = 30) with normal HDL levels were recruited from 2020/03 to 2020/09. Patients with diseases that may affect the function of HDL were excluded, including diabetes, coronary heart disease of stable angina pectoris or unstable angina pectoris, severe trauma, infectious disease, and suffering surgery in recent 3 months. 20 age- and sex-matched healthy subjects were recruited as a control group. All subjects signed informed consent voluntarily, and this study was approved by the ethics committee of Shaanxi Provincial People's Hospital.

### 2.2. HDL Extraction

HDL was extracted according to the method recorded as described [7]: fasting venous blood of healthy subjects and AMI patients on the first day of hospitalization without administration was extracted, then transported to the laboratory at low temperature. After centrifuging (4000 rpm, 4°C, 10 min), upper plasma was obtained. Ethylenediaminetetraacetic acid (EDTA, 134 mM, 1 : 500; Sigma-Aldrich, CAS No: 60-00-4) and 2,6-di-tert-butyl-4-methylphenol (BHT, 200 mM, 1 : 10000; Sigma-Aldrich, CAS No: 128-37-0) were added for antioxidation before the next centrifugation (50000 rpm, 4°C, 21 h). After centrifugation, plasma lipoprotein was stratified due to their density difference: chyle particles (the top layer, milky white), water (the second layer), low-density lipoprotein (LDL, the third layer, orange), HDL (the fourth layer, bright green), and impurities (the bottom layer, orange). Then, the HDL layer was sucked with a sterile syringe (absorbing of LDL and impurities is inevitable) and centrifuged again (50000 rpm, 4°C, 21 h), and then, the HDL layer was absorbed again. Finally, after centrifugation (50000 rpm, 4°C, 21 h) for the third time, HDL was basically purified. After measuring concentration with the BCA protein concentration determination kit (Sigma-Aldrich, CAS No: 71285-M), HDL were put in a refrigerator (4°C) for the next use within 3 weeks. As HDL from one patient was not enough to finish our experiments, plasma of several patients was combined to isolate HDL.

### 2.3. Measurement of HDL Proinflammatory State

HDL proinflammation was determined by a cell-free assay as previously described [7]: Add the equal quality of HDL from each group to a 384-well plate; then, 15 *μ*l of CuCl_2_ (21.7 *μ*mol/l; Sigma-Aldrich, CAS No: 7447-39-4) was added to HDL (including blank) and reaction at 37°C in SpectraMax M5/M5e multidetection reader for 1 hour. Then, 10 *μ*l dichlorofluorescein diacetate (DCFH-DA, dissolved in methanol, 0.2mg/ml; Sigma-Aldrich, CAS No: 4091-99-0) was added to each well before putting the 384-well plate in SpectraMax M5/M5e multidetection reader to record for 12 h.

### 2.4. Proliferation

HUVEC (cultured in endothelial cell medium (ECM, ScienCell, Cat. No. 0503) supplemented with 1% growth factors, 1% penicillin/streptomycin, and 5% fetal bovine serum (FBS)) were planted in a 96-well plate until the cell density was about 50%; ECM containing 1% FBS with or without PD98059 (20 *μ*M, inhibitor of ERK1/2, Sigma-Aldrich, CAS No: 167869-21-8) preincubation for 30 min before HDL (100 *μ*g/ml, equal to 0.26mmol/l) from each group were added. After incubation for 24 h, cells were washed with PBS for 3 times. Then, cells were incubated with cc-k8 (cell proliferation assay kit, Sigma-Aldrich, CAS No: 634847) for 2 hours. Finally, the absorbance (450 nm) OD value of the 96-well plate was obtained by the microplate reader to indirectly reflect the cell proliferation.

### 2.5. Migration

HUVEC were planted in a 24-well plate until the cell density was about 95%; a line was drawn in the middle of the cell (along the diameter of the 24-well plate) with a 200 *μ*l pipette nozzle (the cells are scraped off by the pipette nozzle, and a “road” with missing cells can be seen under the microscope). After washing the cell debris with PBS, ECM containing 1% FBS with or without PD98059 (20 *μ*M, inhibitor of ERK1/2) preincubation for 30 min before HDL (100 *μ*g/ml, equal to 0.26mmol/l) from each group were added. Photos were taken and the original “road” width of each hole was recorded before incubating the cells for 12 h. After 12 h, the width of the “road” was photographed again to compare the current width with the original width to obtain a percentage for analysis.

### 2.6. Angiogenesis

According to the previous description [8], an equal amount of precooled DMEM (high-glucose, Sigma-Aldrich, CAS No: D0819) with matrix gel (Sigma-Aldrich, CAS No: E1270) was mixed and added to the precooled 48-well plate. After shaking gently, the 48-well plate was moved into the cell incubator for 20 min to solidify it. Then, 300 *μ*l HUVEC (cell concentration 10^5^/ml) with or without PD98059 (20 *μ*M, inhibitor of ERK1/2) were added to a 48-well plate and incubated for 30 min. Then, after HDL (100 *μ*g/ml, equal to 0.26 mmol/l) from each group were added and incubated for 12 h, the 48-well plate was photographed (400x), and the data were recorded for analysis.

### 2.7. Western Blot Analysis

HUVEC were planted in 6-well plates until the cell density was about 95%; ECM containing 1% FBS with or without PD98059 (20 *μ*M, inhibitor of ERK1/2) preincubation for 30 min before HDL (100 *μ*g/ml, equal to 0.26 mmol/l) from each group were added. After incubation for 1 h, cell protein was harvested to detect ERK (Cell Signaling Technology, Cat. No. 9102) and its phosphorylation (Cell Signaling Technology, Cat. No. 9101) sites by Western blot.

### 2.8. Statistical Analysis

All data are analyzed with SPSS 20.0 software and mapped by GraphPad Prism 5.0 software. All data are listed as the mean ± standard deviation. An independent-sample *T*-test was used for normally distributed continuous variable comparison and the Mann–Whitney *U*-test for nonnormally distributed continuous variable comparison. An independent-sample *t*-test was used for comparison between two groups and one-way analysis of variance for multigroup comparison. Differences were considered significant when *P* < 0.05.

## 3. Results

### 3.1. Clinical Data

Except for the TC and LDL levels, all clinical characteristics were similar between the healthy subjects and AMI patients (Table 1).

### 3.2. HDL Antioxidant Capacity Detection

**Figure 1:**
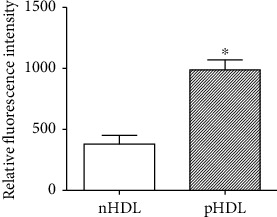
Antioxidant capacity of HDL. The relative fluorescence intensity of pHDL obviously increased (988.83 ± 102.78, *n* = 30) compared with nHDL (379.95 ± 68.52, *n* = 20). High levels of relative fluorescence intensity represent weaker antioxidant capacity. Data are means ± SD, ^∗^vs. nHDL, *P* < 0.05.

The relative fluorescence intensity is negatively correlated with antioxidant capacity. Compared with nHDL (379.95 ± 68.52, *n* = 20) from the control group, the relative fluorescence intensity of pHDL (988.83 ± 102.78, *n* = 30) from the experimental group increased significantly (*P* < 0.05, Figure 1).

### 3.3. Effect of the HDL on Proliferation

**Figure 2:**
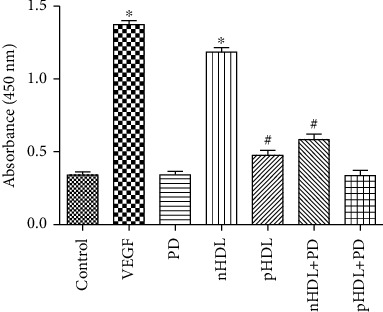
Both VEGF and nHDL can stimulate HUVEC proliferation; pHDL can only slightly stimulate HUVEC proliferation. Preincubation of PD98059 (inhibitor of ERK1/2) can block the effect of HDL on HUVEC proliferation. Data are means ± SD, ^∗^vs. control; ^#^vs. nHDL, *P* < 0.05.

Compared with the control group, both VEGF and nHDL from the control group can stimulate HUVEC proliferation (*P* < 0.05); pHDL from the experimental group can only slightly stimulate HUVEC proliferation (*P* < 0.05), while the ability on stimulating HUVEC proliferation of HDL can be partly blocked by PD98059 (inhibitor of ERK1/2, Figure 2, *P* < 0.05).

### 3.4. Effect of the HDL on Migration

**Figure 3:**
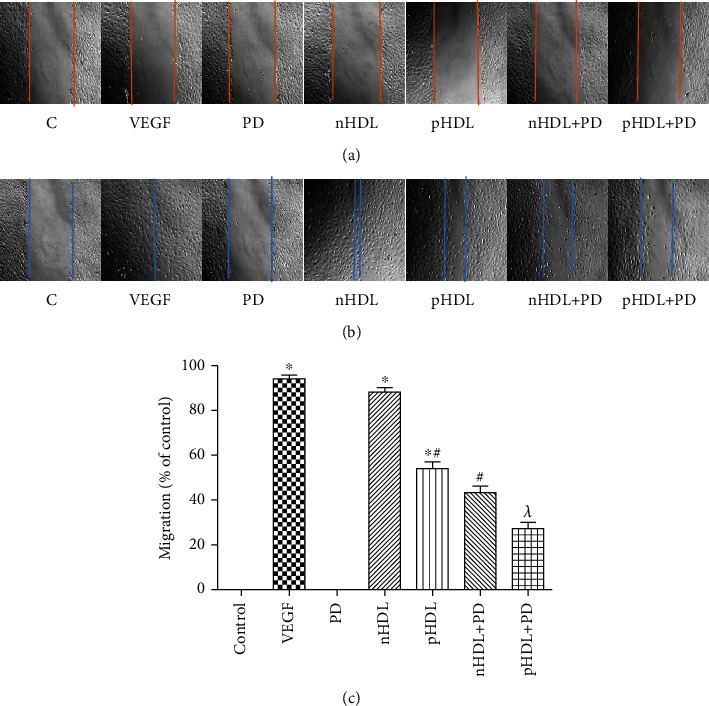
(a) Migration without stimulation and incubation; (b) migration after incubation with HDL, VEGF, and PD98059; (c) percentage of migration conversion. Both VEGF and nHDL can stimulate HUVEC migration; pHDL can only slightly stimulate HUVEC migration. Preincubation of PD98059 (inhibitor of ERK1/2) can partly block the effect of HDL on HUVEC migration. Data are means ± SD, ^∗^vs. control; ^#^vs. nHDL; ^*λ*^vs. pHDL, *P* < 0.05.

Although both VEGF and HDL can stimulate HUVEC migration, the ability on stimulating HUVEC migration of pHDL from the experimental group was weaker than nHDL from the control group (*P* < 0.05). And the ability on stimulating HUVEC migration of HDL can be partly blocked by PD98059 (inhibitor of ERK1/2, Figure 3, *P* < 0.05).

### 3.5. Effect of the HDL on Angiogenesis

**Figure 4:**
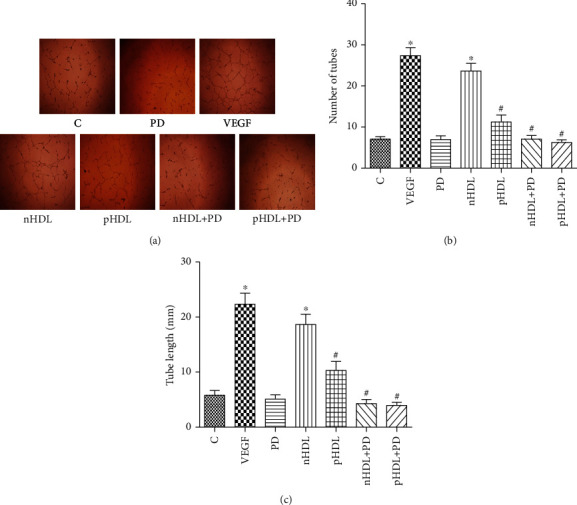
(a) Original image of angiogenesis by matrix gel; (b) number of vessels converted from angiogenesis image; (c) conversion of angiogenesis to vessel length. Although VEGF and HDL can stimulate HUVEC angiogenesis, the ability to stimulate HUVEC angiogenesis of pHDL was weaker than nHDL. Preincubation of PD98059 (inhibitor of ERK1/2) can partly block the effect of HDL on HUVEC migration. Data are means ± SD, ^∗^vs. control; ^#^vs. nHDL, *P* < 0.05, *n* = 8.

Both VEGF and nHDL from the control group can stimulate HUVEC angiogenesis. However, the ability on stimulating HUVEC angiogenesis of pHDL from the experimental group was weakened (*P* < 0.05). The ability on promoting angiogenesis of HDL from both groups can be partly blocked by the inhibitor of ERK1/2 (PD98059, Figure 4, *P* < 0.05).

### 3.6. Effects of HDL on the ERK Expression

**Figure 5:**
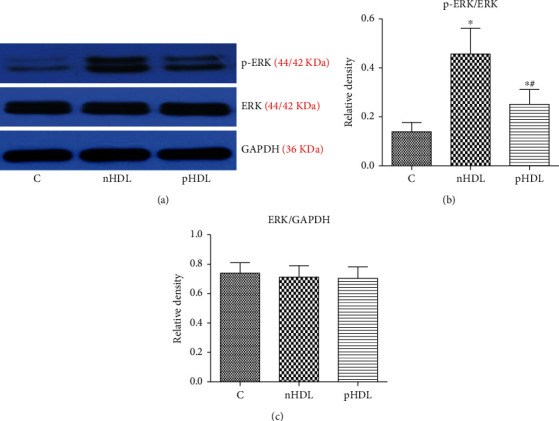
(a) HDL from both healthy subjects (nHDL) and AMI patients (pHDL) can stimulate ERK phosphorylation, but the ability of pHDL was weaker than nHDL. (b, c) The relative density of film. ^∗^*P* < 0.05 vs. (c); ^#^*P* < 0.05 vs. nHDL, *n* = 8.

HDL from both groups can upregulate phosphorylation of ERK. Compared with the nHDL from the control group, pHDL from the experimental group can slightly incubate phosphorylation of ERK (*P* < 0.05, Figure 5).

## 4. Discussion

This study demonstrated that compared with HDL from healthy (nHDL), the antioxidant capacity of HDL from AMI patients (pHDL) was weaker; the ability of pHDL on proliferation, migration, and angiogenesis was weakened. HDL plays its vasoprotective role through the ERK signal pathway.

Vascular endothelial growth factor (VEGF), which plays an excellent role in animal models [9], cannot achieve its expected efficacy when applied to clinical practice [10, 11]. After carefully studying the reports of previous studies, we found that the reports of successful cases in the past were all caused by the application of growth factors after the establishment of the myocardial ischemia model with normal animals [12, 13]. However, most patients with coronary atherosclerotic heart disease in clinical practice are atherosclerosis caused by hyperlipidemia. Some components in hyperlipidemia or substances may affect the therapeutic effect of VEGF on myocardial ischemia or myocardial angiogenesis in myocardial infarction.

HDL is a mixture of protein and lipid with antiatherosclerotic effects. Dalcetrapib, as a cholesteryl ester transfer protein (CETP) inhibitor, can effectively increase the content of HDL. However, the continuous clinical randomized controlled studies suggest that although the level of HDL in patients is significantly increased after the application of dalcetrapib, this change does not reduce the corresponding complications and prevalence of cardiovascular diseases [14]. In the condition that antiangiogenesis prevails disease, such as diabetes mellitus type 2 [9, 15] that is always concomitant with coronary heart disease, whether the vasoprotective function of HDL was affected was unknown. However, the vasoprotective function of HDL (pHDL) is much weaker than normal HDL (nHDL). What is more, HDL in the state of disease also shows signs of cardiovascular damage. For example, the HDL of diabetes mellitus damages the function of endothelium-dependent vasodilation [13].

HDL has an impaired ability to activate endothelial nitric oxide synthase (eNOS) and produce nitric oxide (NO) in patients with coronary heart disease [16]. NO is mainly produced by nitric oxide synthase (NOS) with L-arginine as substrate. A large number of studies have confirmed that NO is one of the indispensable substances for angiogenesis, and NO can also promote angiogenesis [17, 18]. However, the specific mechanism of HDL affecting angiogenesis in patients with coronary heart disease is still unknown. Angiogenesis includes the proliferation, migration, and final differentiation of endothelial cells into blood vessels, and this process also includes the participation of various signal pathways and growth factors [19]. The activation of the ERK signaling pathway is involved in the whole process of angiogenesis (including endothelial cell proliferation, migration, and differentiation or angiogenesis), and studies have shown that ERK can be used as a target for the treatment of angiogenesis [20, 21]. ERK has also been shown to be one of the signaling pathways of VEGF stimulating angiogenesis [22, 23]. Therefore, HDL in normal (nHDL) and AMI patients (pHDL) were tested in the present study. By comparing their effects on HUVEC tube formation and ERK1/2 phosphorylation, we found that nHDL promotes HUVEC tube formation by stimulating ERK1/2 phosphorylation, while the ability of pHDL on HUVEC tube formation and ERK1/2 phosphorylation was weakened.

In summary, our study indicated that the ability of HDL from patients with AMI on tubular formation of HUVEC was weakened resulting from the attenuating ability of phosphorylation of ERK1/2. This result further clarifies that the function of HDL other than its concentration plays a more important role in its vasoprotective function.

## Figures and Tables

**Table 1:** Clinical characteristics.

	Healthy (*n* = 20)	AMI (*n* = 30)
Age (yr)	48.66 ± 7.41	46.58 ± 9.39
Gender, male/female	10/10	14/16
TC (mmol/l)	3.65 ± 0.47	4.71 ± 0.72^∗^
TG (mmol/l)	1.21 ± 0.42	1.34 ± 0.58
HDL (mmol/l)	1.28 ± 0.76	1.21 ± 0.87
LDL (mmol/l)	3.14 ± 0.86	4.22 ± 0.93^∗^
BMI (kg/m^2^)	21.24 ± 1.63	22.23 ± 1.95

Values are listed as means ± SD. TC: total cholesterol; TG: triglyceride; HDL: high-density lipoprotein; LDL: low-density lipoprotein; BMI: body mass index. ^∗^vs. healthy, *P* < 0.05.

## Data Availability

The data that support the findings of this study are available from the corresponding author, Feng-Jun Chang, upon reasonable request.
